# Study protocol for a randomised controlled trial of insulin delivery by continuous subcutaneous infusion compared to multiple daily injections

**DOI:** 10.1186/s13063-015-0658-5

**Published:** 2015-04-16

**Authors:** Jo Blair, John W Gregory, Dyfrig Hughes, Colin H Ridyard, Carrol Gamble, Andrew McKay, Mohammed Didi, Keith Thornborough, Emma Bedson, Lola Awoyale, Emma Cwiklinski, Matthew Peak

**Affiliations:** Department of Endocrinology, Alder Hey Children’s NHS Foundation Trust, Liverpool, L12 2AP UK; Department of Child Health, School of Medicine, Cardiff University, Cardiff, CF14 4XN Wales UK; Centre for Health Economics and Medicines Evaluation, Bangor University, Bangor, LL57 2PZ UK; Liverpool Clinical Trials Research Centre, Liverpool, L12 2AP UK; Department of Diabetes, Alder Hey Children’s NHS Foundation Trust, Liverpool, L12 2AP UK; Department of Research, Alder Hey Children’s NHS Foundation Trust, Liverpool, L12 2AP UK; School of Health, University of Central Lancashire, Lancaster, PR1 2HE UK

**Keywords:** Type I diabetes, Child, Infant, Insulin pump, Multiple daily insulin injections, Randomised controlled trial

## Abstract

**Background:**

Intensive insulin therapy with continuous subcutaneous insulin infusion (CSII) devices or multiple daily injections (MDI) reduces the risk of long-term vascular complications of type I diabetes (TID). Both treatments are used routinely, but there is little evidence to demonstrate superiority of either treatment. If CSII treatment reduces the risk of long-term complications or is associated with an improved quality of life (QoL), the additional cost of this therapy may be compensated for by a reduction in long-term health expenditure. If there is no demonstrable difference between treatments, health-care resources may be better invested elsewhere. This study aims to address this gap in knowledge.

**Methods/design:**

This is a pragmatic, randomised controlled trial (RCT). Fifteen centres, selected to represent a population with a broad demographic, will recruit 316 patients, newly diagnosed with TID, aged between 7 months and 15 years. Exclusion criteria include additional pathologies or treatments likely to affect glycaemic control and a first-degree relative with TID. Randomisation to CSII or MDI is stratified for age, gender and recruiting centre. The randomised treatment starts within 15 days of diagnosis. Patients will be trained to adjust their insulin dose according to carbohydrate intake and blood glucose level.

Study visits coincide with routine clinic appointments at 3, 6, 9 and 12 months when data relating to routine clinical assessments, adverse events and concomitant medications are collected. Health utilities questionnaires are completed at each visit and a diabetes-specific QoL questionnaire (PedsQL) at diagnosis, 6 and 12 months.

The primary outcome is glycaemic control (HbA1c) at 12 months. Secondary outcome measures include QoL, insulin use, growth and weight gain, adverse events and a health economics appraisal.

**Discussion:**

This is the first adequately powered RCT comparing CSII and MDI in a non-selected population, treated according to standard practice guidelines. It will produce data that are meaningful to individual patients and local and national policymakers.

**Trial registration:**

The study was registered with the European Clinical Trials Database on 4 November 2010, reference 2010-023792-25.

## Background

Type I diabetes mellitus (TID) is a common disease of childhood with more than 25,000 children and young people being affected in the United Kingdom [1]. The incidence of childhood TID in Europe is increasing by 3% to 4% a year, with the greatest increases being seen in patients aged less than 5 years. It is predicted that the European incidence of childhood TID will increase from 15,000 in 2005 to 24,400 in 2020, with a doubling in numbers in children aged less than 5 years [2].

The psychosocial and financial cost of TID for patients, the National Health Service (NHS) and society is considerable and is likely to increase. While the prevention and cure of TID remains elusive, it is essential that insulin therapies are used in the most effective and efficient manner.

The optimal treatment for TID would be compatible with the best possible quality of life (QoL) for patients, minimise the risk of acute and long-term complications, and represent good value for money to the NHS. The risk of developing long-term complications is strongly related to the duration of TID and glycaemic control [3]. There is also convincing evidence that intensive insulin regimes, in the form of multiple daily injections (MDI) and continuous subcutaneous insulin infusions (CSII) or insulin pumps, confer an additional benefit in the reduction in risk of long-term complications, over and above improvements in glycaemic control [3]. These methods of insulin therapy are now standard practice in the NHS, although the use of CSII is less prevalent than in other countries [4]. However, there is very little evidence to indicate whether one treatment is superior to the other [5]. The additional cost of insulin pump therapy is estimated to be £1,700 per annum per patient [6]. If this therapy is associated with an improvement in glycaemic control or other measures of health and well-being, the additional cost of this therapy may be offset by a reduction in the complications of TID. On the other hand, if there is no significant difference between CSII and MDI, this investment may be more appropriately directed to other interventions and resources.

In observational studies, glycaemic control [7-11] and QoL or treatment satisfaction [7,8] are reported to improve following the introduction of CSII and the frequency of severe hypoglycaemia is reported to fall [8,9,12]. For these reasons the National Institute for Health and Care Excellence (NICE) recommends insulin pump therapy as a treatment option for patients aged less than 12 years from diagnosis of TID, and for those aged 12 years and above with inadequate glycaemic control on MDI or disabling hypoglycaemia [13]. Approximately 19% of paediatric patients with TID living in England and Wales are treated with CSII [4].

However, when patients are assigned to treatment with either MDI or CSII in the context of a randomised controlled trial (RCT), the beneficial effect of CSII therapy is less convincing. A Cochrane review reporting data from seven paediatric RCTs [14] concluded that HbA1c was 0.2% lower in patients treated with CSII compared with those treated with MDI (95% confidence interval (CI): 0.40% to 0.10%; *P* = 0.02). Six paediatric RCTs, including three studies included in the Cochrane review, were included in a recent meta-analysis that also concluded that CSII had a favourable effect on HbA1c of 0.24% (95% CI: −0.41 to −0.07; *P* < 0.001), and required lower doses of insulin than MDI (insulin dose 0.22 units per kg per day lower, 95% CI: 0.31 to 0.14; *P* < 0.001) [15]. However, there was no significant difference in the rate of severe hypoglycaemic events or diabetic ketoacidosis. A meta-analysis of QoL was not undertaken in this meta-analysis or the Cochrane review, because the tools used to measure QoL differed between studies.

Although trial evidence suggests a favourable effect of CSII, it is important to note that there are a number of weaknesses in most studies reported to date. Studies that recruit patients with established TID treated with MDI, may show an improvement with insulin pump therapy for two reasons: (1) selection bias, since patients with good glycaemic control who are satisfied with MDI therapy are less likely to be approached or agree to participate in these studies and (2) increased contact with diabetes health-care professionals because at the start of insulin pump therapy patients receive an intensive period of education. Insulin analogues have been reported to improve glycaemic control in paediatric practice [16] though only one study uses analogue insulin for the treatment of all patients using MDI [9]. The number of patients recruited to studies is generally small, between 16 and 72, and these studies are probably underpowered. Children aged less than 5 years are over-represented. Furthermore, there are only two multi-centre studies [17,18], one 2-centre study [19] and three [20-22] of the ten RCTs are single-centre. The observation period is 3.5 months in some studies, and may be too brief for patients to acquire the skills required to use CSII to its full potential [21-23]. In other studies the observation period (4 to 6 months) may be too brief to show patient fatigue in the use of this intensive therapy [20,24-26].

To date no RCT has been delivered in the NHS setting, where health-care services, lifestyle and diet differ from those in which CSII and MDI have been studied previously, and no study has investigated the health economics of CSII.

The SCIPI (**S**ub**C**utaneous **I**nsulin: **P**umps or **I**njections?) study will address these gaps in the current knowledge base, generating data that are generalisable to the population of children and young people with TID, treated within the NHS according to current treatment guidelines. In doing so, we aim to make a valuable contribution to the debate regarding the optimal use of health-care resources for these young patients.

## Methods/design

### Ethical approval

The protocol has been approved by the Liverpool East Research Ethics Committee, reference 10/H1002/80 and the Medicines and Healthcare Products Regulatory Agency (MHRA).

Local approvals have been obtained from Alder Hey Children’s Hospital; Birmingham Children’s Hospital; Doncaster Royal Infirmary; East Surrey Hospital; Ipswich Hospital NHS Trust; Mid Staffordshire Hospital; Norfolk and Norwich University Hospital; Oxford Children’s Hospital; Queen’s Medical Centre, Nottingham; Royal Blackburn Hospital; Royal Preston Hospital; Royal Victoria Infirmary, Newcastle Upon Tyne; Sheffield Children’s Hospital; Southampton General Hospital and University Hospital of Wales, Cardiff. Recruitment to the study at individual sites can only start once local approvals have been obtained.

### Design

**Figure 1:**
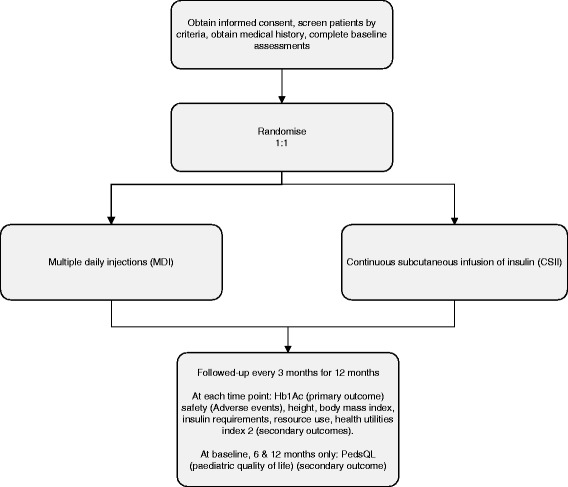
CONSORT flow diagram.

This is a pragmatic, multi-centre RCT with an internal pilot phase, in which insulin delivery by CSII is compared to insulin delivery by MDI in children and young people aged 7 months to 15 years, newly diagnosed with TID. A schematic representation of the study design is given in Figure 1.The internal pilot study, with a sample size of 30 participants, tested the feasibility of recruitment. The study proceeded to the full study phase when the following criteria were met: (a) consent was obtained from more than 50% of eligible patients who were invited to participate and (b) demographic characteristics, including age, ethnicity, gender and deprivation score, were not considered to be significantly different in the group of patients who were recruited compared to those who declined.

### Centre and patient selection

Patients are recruited through 15 centres in the United Kingdom located in Liverpool, Newcastle, Cardiff, Oxford, Birmingham, Nottingham, Southampton, Preston, Doncaster, Norwich, Redhill, Blackburn, Stafford, Sheffield and Ipswich. Centres have been selected to ensure that patients are recruited from a demographically diverse population. To be eligible to participate in the study, centres must have ten or more patients treated with CSII within their clinic population, and have sufficient resources to deliver the clinical aspects of the study protocol. All centres have a study nurse. The inclusion and exclusion criteria are given in Table 1.

**Table 1:** **Participant inclusion and exclusion criteria**

**Inclusion criteria**	**Exclusion criteria**
Newly diagnosed type I diabetes mellitus using standard diagnostic practice	Treated previously for diabetes
Age 7 months to 15 years (inclusive)	Haemoglobinopathy
Parent or legal representative of the patient is willing to give consent for the study	Co-existing pathology conditions likely to affect glycaemic control
Parent or legal representative of the patient is able to comply with the treatment regimen and study visits	Psychological or psychiatric disorders, e.g. eating disorders
Patient aged 8 years and over and is able to adhere to the treatment regimen and study visits	Receiving medication likely to affect glycaemic control
	Allergic to a component of insulin aspart or insulin glargine
	Thyroid in a non-euthyroid state
	Coeliac disease and unable to maintain a gluten-free diet

### Sample size

#### Internal pilot

A total of 30 subjects were recruited providing 80% power to detect a drop in the consent rate to 25% from the assumed 50%, at the 5% significance level.

#### Full trial

The study has been powered to detect a difference in HbA1c between treatment arms of 0.5%, this being the threshold used by the Food and Drug Administration and pharmaceutical industry to determine effectiveness of new oral hypoglycaemic agents . A sample size of 143 in each group has 80% power to detect a difference in mean HbA1c of 0.50%. To allow for a loss to follow-up of 10%, 316 patients will be recruited.

### Patient recruitment

Patients and families are given verbal and written information about the study at the time of diagnosis of TID. Since March 2014, a short film describing participants’ experiences has been available for eligible patients, their parents and careers. Written, informed consent, and where appropriate, assent, is obtained for all study participants. The timing of consent is dependent on the needs and wishes of the patient and family. However, consent must be obtained within a time frame that allows for the randomised treatment to start within 14 days of diagnosis of TID.

### Randomisation

Participants are randomised using a secure web-based randomisation programme. Participants are randomised to each treatment arm in a 1:1 ratio with stratification for age and recruiting centre. The randomisation code list is generated by a statistician using block randomisation with the random variable block size method.

### General procedures and measurements

#### Intervention

Patients are treated according to local clinical practice from the time of diagnosis until the start of randomised treatment.

##### Insulin therapy

Starting insulin regimens are standardised across both treatment arms, and then modified according to clinical need by the treating clinician. Insulin doses are titrated according to blood glucose levels recorded at home. For both treatment arms the total daily dose (TDD) of insulin is calculated from body weight: 0.5 units per kg of body weight per day in pre-pubertal patients and 0.7 units per kg of body weight per day in pubertal subjects. Patients randomised to treatment with MDI administer 50% of the TDD as insulin glargine (a long-acting insulin analogue) once daily. The remaining 50% of the TDD is administered as insulin aspart (a short-acting insulin analogue) in three divided doses, before meals. Further boluses of insulin aspart are given when 10 g or more of carbohydrate are consumed. Patients treated with CSII infuse 50% of the TDD as insulin aspart at a basal rate, with the remaining 50% of the TDD being given as boluses before meals. Additional boluses of insulin aspart are given when 5 g or more of carbohydrate are consumed. Correction doses for the treatment of high blood glucose values for both treatment arms are calculated according to the 100 rule, in which the amount of insulin required to reduce the blood glucose by 1 mmol/L is calculated from the TDD of insulin [27].

##### Education for both treatment arms

At entry to the study, all participants complete a structured educational program delivered to participants and their families in accordance with the standards of the International Society for Paediatric and Adolescent Diabetes [28]. All the participants are trained in the use of MDI and the use of the Roche Expert glucometer, whilst participants randomised to CSII treatment are also trained in the use of CSII pumps.

##### Data collection

Table 2 shows the schedule of study visits, timed to coincide with routine clinic appointments according to the standards of the NICE guideline [29].

**Table 2:** **Study procedures and data collection**

**Procedures**	**Baseline**		**Follow-up: scheduled 3-monthly clinic visit from time of diagnosis**			
	**Diagnosis**	**Prior to start of treatment**				
	**T0**		**T + 3**	**T + 6**	**T + 9**	**T + 12**
**Assessment of eligibility criteria**	X					
**Signed consent form**	X					
**Randomisation**	X					
**Review of concomitant medications**		X	X	X	X	X
**Review of insulin use (insulin requirements)**						
**Patient diaries**			X	X	X	X
**General practitioner prescriptions**			X	X	X	X
**CSII pump download**			X	X	X	X
**Review of medical history**	X			X		X
**Blood glucose measurement**	X^b^	X	X	X	X	X
**Blood pH measurement**	X					
**Demographics**	X					
**Study intervention**		X^a^				
**Physical exam**						
**Height**	X		X	X	X	X
**Weight**	X	X	X	X	X	X
**Injection sites**			X	X	X	X
**Symptom-directed**			(X)	(X)	(X)	(X)
**Assessment of adverse events**			(X)	(X)	(X)	(X)
**Clinical laboratory**						
**HbA1**_**c**_ **(local analysis)**		X	X	X	X	X
**HbA1**_**c**_ **(central analysis)**		X	X	X	X	X
**Chemistry**	(X)					X
**Haematology**	(X)					X
**Urinalysis**	(X)		X	X	X	X
**Patient completed measures**						
**Patient PedsQL**		X		X		X
**Parent PedsQL**		X		X		X
**Health utilities index 2**		X	X	X	X	X
**Resource use: RN completed CRF**		X	X	X	X	X

(X), as indicated/appropriate.

^a^Randomised treatment will be commenced within 14 days of diagnosis; ^b^measurement of blood glucose will be undertaken in the local hospital laboratory at diagnosis and by glucometer at remaining time points. RN: research nurse, CRF: case report form.

A full clinical history is taken at the time of diagnosis of TID. A venous blood sample is collected at study entry for the measurement of electrolytes, urea, pH, glucose and HbA1c. Thyroid-stimulating hormone and free T4 are measured to screen for disorders of thyroid function. Tissue transglutaminase antibodies and immunoglobulin A are measured to screen for coeliac disease. These blood tests are repeated on completion of the study protocol. Details of concomitant medications are collected at each study visit.

#### Procedures for assessing efficacy

##### Primary outcome

The primary outcome of the study is HbA1c 12 months after diagnosis of TID. HbA1c will be measured in the local laboratory and centrally at Alder Hey Children’s NHS Foundation Trust in Liverpool. Samples will be collected at diagnosis and then at 3, 6, 9 and 12 months.

##### Secondary outcomes

Secondary outcome measures include insulin use, growth, weight gain, QoL, adverse events and a health economics assessment.

##### Insulin requirements

Insulin usage data are downloaded from the CSII pumps for participants receiving CSII at each study visit. Participants treated with MDI record the insulin doses in patient-held diaries used as part of routine practice. Data are collected at each study visit. At each follow-up visit the participant’s general practitioner is contacted to ascertain the quantity of insulin prescribed for the participant. This is compared to the quantities recorded by participants to guard against significant over-reporting.

##### Growth and weight gain

Height and weight are measured at diagnosis, and then at the 3-, 6-, 9- and 12-month study visits. Body mass index (BMI) standard deviation score (SDS) are derived from 2007 World Health Organisation growth data [30,31].

##### Adverse events

Details of related adverse events, including episodes of severe hypoglycaemia, diabetic ketoacidosis, lipohypertrophy and injection/ infusion site infections, are collected at each study visit and reported according to the requirements of the MHRA. Adverse events are captured by patients, carers and clinicians treating diabetes, and from interrogation of local hospital databases for details of hospital attendances. Insulin injection sites are examined at every visit for lipohypertrophy.

##### Quality of life

QoL is assessed using validated diabetes-specific QoL (PedsQL) questionnaire instruments [32] prior to start of treatment then at 6 and 12 months after diagnosis. Children and young people aged 5 to 7 years, 8 to 12 years and 13 to 18 years will use developmentally appropriate versions, and the parental version is used for all age groups [33].

##### Health economics analysis

A cost utility analysis will be conducted to estimate the incremental cost per quality-adjusted life year (QALY) gained with CSII versus MDI. The health economics analysis will adopt the perspective of the NHS, and consider costs associated with both treatment arms, CSII (insulin purchase, maintenance and use of disposables) and MDI: insulin use, contact with health professionals and costs associated with adverse events including investigations, procedures, treatments and hospitalisations. Time spent with diabetes health-care practitioners (nurses, medical staff, dieticians and psychologists) will be collected prospectively. Participants’ use of other services will be collected by means of a health resource use questionnaire prior to the start of randomised treatment (for the previous 3 months) then at the 3-, 6-, 9- and 12-month study visits.

Unit cost data will be obtained from reference sources, including routine hospital data (NHS reference costs [34]) and nationally published data [35]. Additionally, each recruiting hospital will provide details of inpatient admissions, including ward name and specialty (e.g. paediatric, paediatric ICU, etc.); the average cost per bed day on the ward and number of occupied bed days on the ward.

QALYs will be estimated from patients’ (12 years and over) and their parents’ or guardians’ responses to the Health Utilities Index Mark 2 questionnaire. The six attributes of this questionnaire (sensation, mobility, emotion, cognition, self-care and pain) will be summarised into a single UK-derived preference-based utility score [36].

### Analysis

#### Primary outcome

The trial will be analysed and reported using the Consolidated Standards of Reporting Trials (CONSORT) and the International Conference on Harmonisation E9 guidelines. A full and detailed statistical analysis plan will be developed prior to any comparative analysis of the trial data. The main features of the statistical analysis plan are included here.

A *P* value of 0.05 or less will be used to declare statistical significance for all analyses. Rather than adjust for multiplicity, relevant results from other studies already reported in the literature will be taken into account in the interpretation of results.

The primary analysis will use the intention-to-treat principle, and a secondary analysis will be conducted using the per-protocol approach. The purpose of the per-protocol approach is to consider the robustness to protocol deviations of the conclusions reached from the analysis using the intention-to-treat principle.

The primary outcome HbA1c will be compared between the trial groups using a two-group *t*-test. Difference in means with 95% confidence intervals will be presented. Analysis of covariance will be used to adjust for baseline values and important prognostic factors.

Missing data will be monitored and strategies developed to minimise its occurrence; however, as much data as possible will be collected about the reasons for missing data and this will be used to inform the handling of missing data.

#### Health economics

Total costs will be calculated as the sum-product of resource use and unit cost for each patient. QALYs will be calculated as the area under the utility-time curve over 12 months. Cost-effectiveness will be determined by the ratio of the differences between treatment groups in mean costs to QALYs. Uncertainty in the incremental cost-effectiveness ratio (ICER) will be addressed through the application of bootstrapping and the estimation of cost-effectiveness acceptability curves [37]. A regression analysis of cost and QALYs, with age, baseline HbA1c, utility, cost [38] and other covariates as deemed appropriate, will be conducted to improve precision in the cost utility estimate while conserving any correlation between costs and benefits. Estimates of ICERs will be compared with the £20,000 to £30,000 per QALY threshold of cost-effectiveness set by NICE. An exploratory analysis in which trial results will be extrapolated to estimate lifetime costs and benefits and to capture long-term micro- and macrovascular complications, will be accomplished by using the CORE diabetes model [39].

## Discussion

This study critically aims to compare the benefits and disadvantages of insulin delivery by MDI and CSII for children and young people. In developing this study protocol, we have considered carefully the strengths and weaknesses of previous studies. We have elected to recruit patients with newly diagnosed TID, and excluded those with affected first-degree relatives, to minimise the bias that is likely to result from a patient’s or family’s previous experience of success or failure of one treatment arm. To ensure that the data generated in this study are generalisable to the population of children and young people with TID, treated in the NHS we have: (1) selected centres with diverse patient demographics, and demonstrated that the characteristics of the population of patients who consent to participate do not differ from those who decline; (2) stated a requirement that patient management is directed by local clinicians according to standard clinical practice and (3) designed study visits to coincide with national guidelines for the timing of clinic visits, thereby eliminating additional patient support and education that would result from a more intensive visit schedule and maximising concordance with the study protocol. A wide range of benefits and disadvantages of MDI and CSII have been reported previously in observational trials and RCTs. In selecting glycaemic control as the primary outcome, we have chosen a parameter that can be robustly measured and may be related to long-term outcomes and costs to patients and health services [40,41]. While QoL is undoubtedly important, we are concerned that the paediatric diabetes QoL questionnaires validated to date may not be sufficiently sensitive to identify the ways in which these modes of insulin delivery impact QoL differently. For this reason QoL is a secondary outcome measure.

In delivering the study we face a number of challenges. Patients have to be recruited shortly after the diagnosis of TID, at a time when they and their families may feel overwhelmed by the diagnosis and unable to consider participation in a clinical trial. Treatment at the time of diagnosis is determined by local practice, and in most centres patients will be treated with MDI. Some patients and families develop a strong preference for this treatment and are reluctant to consider CSII therapy. To try to address this preference, we have made a short film in which patients treated with MDI and CSII share their experiences of their treatment and participation in the study. Screening log data has shown that patients who are approached soon after diagnosis are more likely to consent to participate in the study, than those approached later. We speculate that this may be because patients are less likely to have developed a preference for MDI and are more open to the possibility of treatment with CSII. We have used this information to develop a recruitment strategy that includes an early approach to families. For some centres, this will be their first experience of CSII therapy from the time of diagnosis. There are logistical challenges in educating and supporting patients and families in establishing CSII therapy within the time constraints of the study protocol.

The SCIPI study will be the first RCT of MDI and CSII, delivered in an unselected population and adequately powered to detect a difference in glycaemic control. The protocol has been developed carefully to ensure that the clinical outcomes are meaningful for patients treated in the NHS. We have also addressed the needs of national and local policymakers, by including a range of outcomes important to the development of clinical guidelines, including a robust health economics appraisal. For these reasons we believe this study will make a valuable contribution to the care of children and young people with TID.

## Trial status

The protocol has been approved by the Liverpool East Research Ethics Committee, reference 10/H1002/80, and was registered with the European Clinical Trials Database, reference 2010-023792-25 on 4 November 2010. The study was registered with the ISRCTN, registration number ISRCTN2925527, on 12 November 2010. Site-specific approval has been obtained at all recruiting sites. In total, 250 participants have been recruited.

## Abbreviations

BMI: body mass index
CI: confidence interval
CONSORT: Consolidated Standard of Reporting Trials
CSII: continuous subcutaneous insulin infusion
ICER: incremental cost-effectiveness ratio
MDI: multiple daily injections
MHRA: Medicines and Healthcare Products Regulatory Agency
NHS: National Health Service
NICE: National Institute for Health and Care Excellence
QALY: quality-adjusted life year
QoL: quality of life
RCT: randomised controlled trial
TDD: total daily dose
TID: type I diabetes mellitus
